# MISCLASSIFICATION IN DEMENTIA DATA IN THE CARDIOVASCULAR HEALTH STUDY: A PROBABILISTIC BIAS ANALYSIS

**DOI:** 10.1093/geroni/igz038.2879

**Published:** 2019-11-08

**Authors:** Michelle Odden, Alice Arnold, Anne B Newman, Adina Zeki Al Hazzouri, Lindsay Miller, Oscar Lopez, Kenneth Mukamal, Andreea Rawlings

**Affiliations:** 1 Stanford University, Stanford, California, United States; 2 University of Washington, Seattle, Washington, United States; 3 University of Pittsburgh, Pittsburgh, Pennsylvania, United States; 4 Columbia University, New York, New York, United States; 5 Oregon State University, Corvallis, Oregon, United States; 7 Beth Israel Deaconess Medical Center, Boston, Massachusetts, United States; 8 Kaiser Permanente Center for Health Research, Portland, Oregon, United States

## Abstract

Data routinely collected in observational studies from phone calls, medical records, and death certificates can be used to categorize dementia, though there may be misclassification. We applied probabilistic bias analyses to assess the magnitude, direction, and uncertainty of the error due to misclassification of dementia data in the Cardiovascular Health Study (CHS). We categorized dementia among all participants (73,284 person-years) using medications, ICD-9 codes, use of proxy, and death certificates, and compared to the gold standard adjudicated dementia in the CHS Memory Studies (28,250 person-years). Using the gold standard, positive (PPV) and negative predictive values (NPV) of dementia categorization were estimated within strata defined by age and sex. In probabilistic bias analyses, we reclassified participants from the full study using estimated PPVs and NPVs in 5,000 replicates. We estimated the hazard ratio (HR) of dementia associated with age, race, sex, hypertension, diabetes, and APOE4 genotype in this bias analysis and compared these results to those using original data. ICD-9 codes had low specificity and were excluded in further analyses. The NPV was differential by sex (66% for females and 79% for males) and race (51% for blacks, 60% for whites). In bias analysis, the HR for black race was attenuated from 2.81 (95%CI:1.36-5.80) to 1.23 (95%CI:1.14-1.33). The estimate for hypertension was statistically significant only in bias analysis. Estimates and inferences for the other covariates were modestly different. Differential misclassification may lead to important biases of risk factors, but can be recognized and addressed using probabilistic bias analyses.

